# Opposites in Reasoning Processes: Do We Use Them More Than We Think, but Less Than We Could?

**DOI:** 10.3389/fpsyg.2021.715696

**Published:** 2021-08-26

**Authors:** Erika Branchini, Elena Capitani, Roberto Burro, Ugo Savardi, Ivana Bianchi

**Affiliations:** ^1^Department of Human Sciences, University of Verona, Verona, Italy; ^2^Department of Humanities (Philosophy and Human Sciences Section), University of Macerata, Macerata, Italy

**Keywords:** opposites, contrast class, inductive and deductive reasoning, insight problem solving, counterfactual thinking

## Abstract

Our aim in this paper is to contribute toward acknowledging the general role of opposites as an organizing principle in the human mind. We support this claim in relation to human reasoning by collecting evidence from various studies which shows that “thinking in opposites” is not only involved in formal logical thinking, but can also be applied in both deductive and inductive reasoning, as well as in problem solving. We also describe the results of a series of studies which, although they have been developed within a number of different theoretical frameworks based on various methodologies, all demonstrate that giving hints or training reasoners to think in terms of opposites improves their performance in tasks in which spontaneous thinking may lead to classic biases and impasses. Since we all possess an intuitive idea of what opposites are, prompting people to “think in opposites” is something which is undoubtedly within everyone's reach and in the final section, we discuss the potential of this strategy and suggest possible future research directions of systematic testing the benefits that might arise from the use of this technique in contexts beyond those tested thus far. Ascertaining the conditions in which reasoners might benefit will also help in terms of clarifying the underlying mechanisms from the point of view, for instance, of analytical, conscious processing vs. automatic, unconscious processing.

## Introduction

Humans have an intuitive idea of opposites in addition to an intuitive idea of what constitutes similarity, diversity, and sameness. Same-different tasks have been extensively used in Psychology to study perception and categorization without the need to explain to participants what the terms “same” or “different” mean. We are all familiar with the experience of needing to change an item we have just bought when we notice a defect and we expect the salesperson in the shop to exchange it with an identical item (that is, the same size, color, and design etc.). If the assistant tries to give us a different item or even a similar one, he/she will need to convince us to accept it as a replacement since we see it is not the same as the original item. In the same way, we immediately recognize whether two sounds are identical, similar or different in terms of a series of opposite features, for example, high-low, increasing-decreasing, regular-irregular or pleasant-unpleasant. These are all understood by us as being opposite attributes without the need for any formal definition. These might seem to be quite trivial examples, but their obviousness shows us that we readily acknowledge how basic and pervasive these relationships are in the structure of our everyday lives.

**Figure 1:**
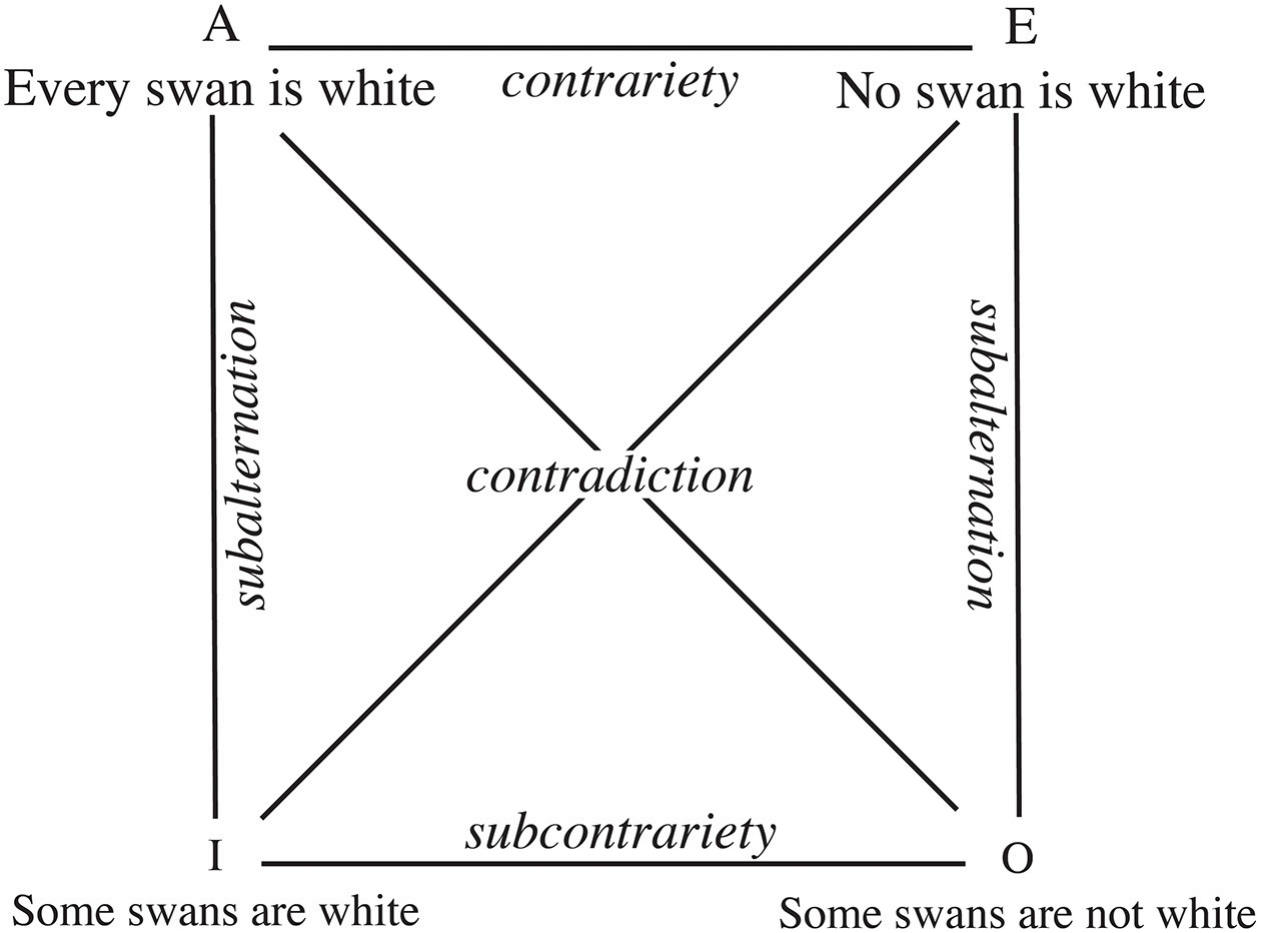
The square of opposition refers to a diagram which was introduced in traditional logics. It represents four types of logical relationships holding between four basic forms of propositions: universal affirmatives (A), universal negations (E), particular affirmatives (I), and particular negations (O).

The idea that similarity is transversal to many different cognitive functions has been accepted in Psychology at least since Tversky's seminal work (Tversky, 1977). “Similarity is a central construct in cognitive science. It is involved in explanations of cognitive processes as diverse as memory retrieval, categorization, visual search, problem solving, induction, language processing, and social judgment (see Hahn et al., 2003, and references therein). Consequently, the theoretical understanding of similarity affects research in all of these areas” (Hahn et al., 2009). Any cognitive scientist engaged in describing how human perception, categorization, language or reasoning works will continuously come across similarity as a key concept in the organizing principle by means of which individuals classify objects, form concepts, and make generalizations (for a review, see Goldstone, 1994). We much less frequently hear cognitive scientists making references to opposites when discussing the same processes. Our aim with the present paper is to encourage cognitive scientists that the role of opposites in cognition should not be relegated to formal logical matters such as those traditionally exemplified by “the square of opposition” (Figure 1; see Beziau and Basti, 2016 for an update on the developments of the square of opposition within modern logics).

In the following sections of the present paper (sections Thinking in Terms of Opposites in Order to Figure Out Alternatives in Everyday Life, Opposites and Deductive Reasoning, Opposites and Inductive Reasoning, and Opposites and Insight Problem Solving) we draw attention to the role that opposites play in reasoning processes such as everyday counterfactual thinking, classic deductive and inductive reasoning tasks and the representational changes required in certain reasoning tasks. An inspection of the contexts in which opposites are implied in spontaneous human thinking and of others in which they are crucially needed (even though not spontaneously applied) suggests that the notion of opposites supports human thinking in a number of ways. If we acknowledge this, it follows that opposites can be regarded as a general organizing principle for the human mind rather than simply a specific relationship (however respectable) merely related to logics. This broader perspective on opposites which sees them as useful in a variety of reasoning processes leads to new questions and suggests new directions for research, as we propose in the final section of the paper (section Discussion). Before addressing all these points, we refer to three different approaches which have been developed within the field of Cognitive science in the last 20 years that—although in different ways—all deem opposites to be a general and basic phenomenon. What is fundamental to these approaches is the idea that opposites are a primal organizing principle for the human mind which applies to language, perception and relational reasoning.

**Figure 2:**
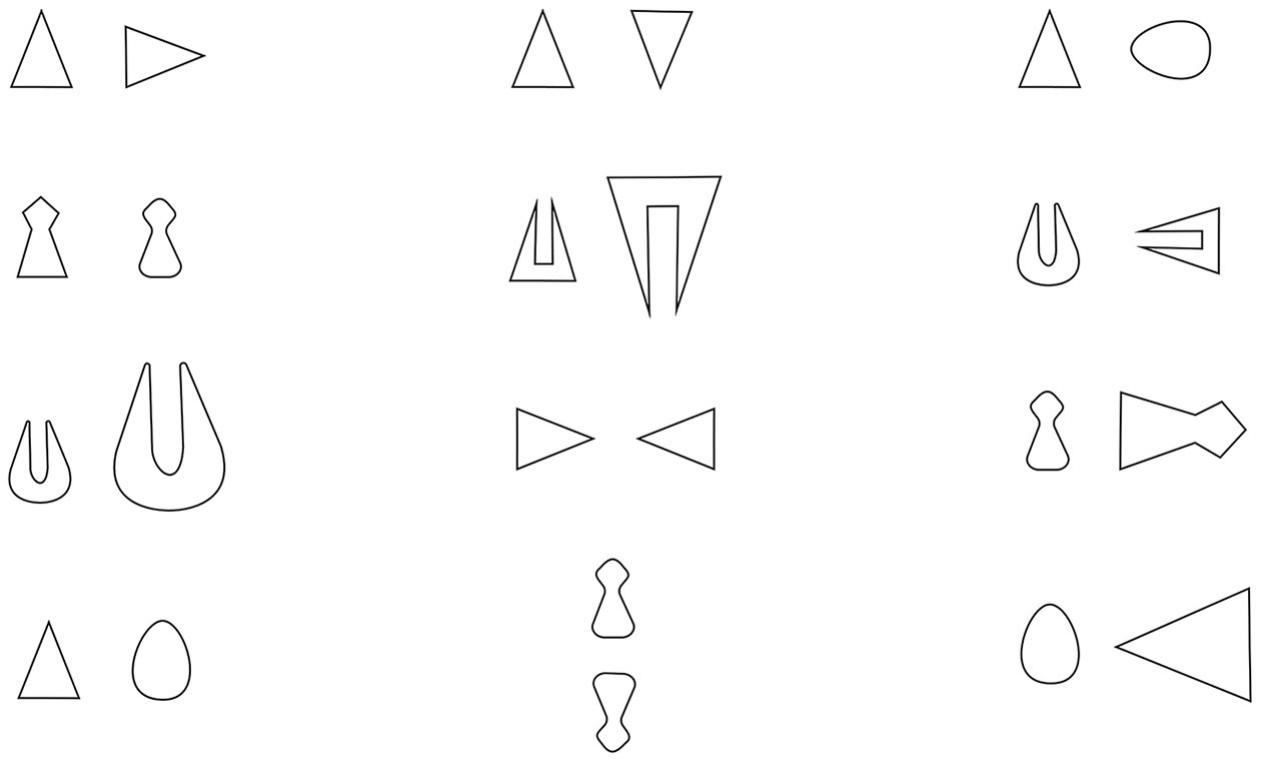
Some examples of pairs of configurations formed of two simple bidimensional figures which are perceived as similar (1st column), opposite (2nd column), and different (3rd column)—Bianchi and Savardi (2008a).

(i) Cognitive linguists have noted and discussed the pervasiveness of antonyms in *natural human languages* (see, for instance, Jones, 2002, 2007; Paradis et al., 2009). It has been acknowledged that opposites represent a special semantic relationship which is more easily learned and more intuitively understood than any other semantic relationship and indeed non-experts can easily identify opposites, despite being unable to formulate a clear definition of the requisite for two meanings to be opposites (e.g., Kagan, 1984; Miller and Fellbaum, 1991; Fellbaum, 1995; Jones, 2002; Murphy, 2003; Croft and Cruse, 2004). It has been shown that this primary intuition has its roots in infants' pre-verbal categorization (e.g., Casasola et al., 2003; Casasola, 2008).

(ii) A second approach involves the hypothesis that opposites are a specific, basic, *perceived relationship*. This approach (the main theoretical and methodological framework of which are summarized in Bianchi and Savardi, 2008a) has led to various experimental explorations of the abilities of adults and children to recognize whether certain configurations are “opposite” as compared to “different” or “similar” and to also produce opposite configurations. These explorations referred to various perceptual domains and contents and involved, for example, simple visual configurations (e.g., Bianchi and Savardi, 2006; Schepis et al., 2009; Savardi et al., 2010; Bianchi et al., 2017a), everyday objects and/or environments (e.g., Bianchi et al., 2011, 2013, 2017c), human body postures (e.g., Bianchi and Savardi, 2008b; Bianchi et al., 2014), and acoustic stimuli (e.g., Biassoni, 2009; Bianchi et al., 2017b). The results emerging from these studies indicated that (a) the participants were consistent in defining the point along a dimension at which a property stops being perceived as pertaining to one extreme of the dimension (e.g., *near* on the dimension *near/far*) and starts to be perceived as an instance of the opposite property (e.g., in this case *far*) or of an intermediate region with variations that are not perceived as pertaining to either one pole or the other (e.g., *neither near nor far*); (b) two configurations are perceived to be opposites when they show a maximum contrast in terms of a perceptually salient property—usually regarding the orientation or direction of the configuration—within a condition of otherwise overall invariance (some examples are provided in Figure 2); and (c) due evidently to this close connection between the perception of opposition and the perception of opposite orientation in an overall invariant configuration, opposition is also perceived in configurations involving mirror symmetry, including people's perception of their own body reflection in a plane mirror.

(iii) A third approach focuses on people's ability to discern three different types of oppositional relationships between objects, concepts and ideas, that is anomaly, antinomy, and antithesis. The recognition of deviance (anomaly), incompatibility (antinomy), and opposition (antithesis) has been shown to be critical not only for complex thought (Chinn and Brewer, 1993; Holyoak, 2012), but also with regard to professional abilities in medicine (Dumas et al., 2014), chemistry (Bellocchi and Ritchie, 2011), and engineering (Dumas and Schmidt, 2015; Dumas et al., 2016). This approach was developed over the course of a decade in relation to a number of domains (see Alexander, 2012; Dumas et al., 2013; Alexander et al., 2016) and a Test of Relational Reasoning (TORR) was developed to measure the aforementioned forms of relational reasoning, and also analogy which refers to thinking in terms of similarity rather than opposition (Hesse, 1959; Goswami and Mead, 1992; Alexander et al., 1997; Dunbar, 2001; Hofstadter, 2001; Gentner et al., 2003; Braasch and Goldman, 2010). The anomalous reasoning scale of the TORR gauges respondents' ability to discriminate a deviance or a discrepancy by recognizing an object that does not fit a given or typical pattern. The antinomous reasoning scale measures the ability to reason with mutual exclusivity between categories and to recognize a paradoxical situation (i.e., a situation in which two conditions cannot both be true). The antithetical reasoning scale measures the ability to recognize a direct oppositional relation between situations. The TORR measures the ability to recognize these relationships both with regard to visual stimuli and to written sentences.

This study focuses on the role of opposites in relation to *various types of reasoning tasks*. In this sense, we come closer to the third approach described in the previous section (point iii above). However, the phenomenon is observed from a standpoint which is less technical than that presupposed by that approach and is more similar to the intuitive idea of opposition relating to the other two approaches (that is points i and ii above). Indeed, when we refer to “thinking in opposites,” we do not refer to any technical or logical definition which, conversely, constructs such as antithesis, contradiction, and counterfactuality necessarily imply. We start, more simply, from the consideration that when humans conceive of a variation from a given state, object, action or situation, they necessarily do it with reference to opposites. For the sake of intellectual honesty, it is to be noted that the roots of this idea were already present in the work of Aristotle (1984, Cat. 5, 4a 30–34), but have been explicitly applied to the human cognitive system in more recent times (e.g., Gärdenfors, 2000, 2014; Paradis, 2005; Bianchi and Savardi, 2008a). By saying that every kind of perceptually identifiable variation between two objects relates in some way to opposition, we do not mean that the final outcome is necessarily globally perceived as opposite; from a global perspective, two objects might simply appear diverse or similar. This depends on the number of features which are transformed and on which features are transformed. Indeed, in the case of similarity, diversity and opposition, both the number of common, distinctive features and the salience of those features are critical factors (the literature supporting this statement is reviewed in Bianchi and Savardi, 2008a, p. 129–130). However, in order to stand up to an analytical (i.e., local) inspection, any variation must necessarily consist of a change from one property or state to another property or state along a dimension which is cognitively defined by two opposite properties. This can involve varying degrees of alteration, for example, something small can be transformed either into something big (i.e., its opposite) or into which is still perceived as small but is less small than before. The transformation may also comprise a variation which shifts toward an intermediate property (e.g., something small becomes something which is perceived as neither small nor big).

The fact that there is a close link between non-identity and opposition has also emerged in research investigating the extent to which the negation of one property (e.g., not good) implies affirming the opposite property (e.g., bad). For instance, “The water is not hot” makes us think of water than might be warm, lukewarm or cool. It has been empirically proved that inferring one of these options in preference to the others depends in part on rhetoric aspects (e.g., Colston, 1999; Horn and Kato, 2000) and in part on semantic (e.g., Paradis and Willners, 2006) and perceptual aspects (e.g., Bianchi et al., 2011) relating to both the nature of the negated property itself and the opposite pole. In any case, understanding negation seems to entail a mental construction of a model implying a variation from the negated situation identified within a dimension with two opposite poles (Kaup et al., 2006, 2007).

This idea that every variation (including negation) which occurs along a dimension with opposite poles applies to the present paper in the sense that we see that “thinking in opposites” becomes the natural background for creating mental *alternatives*. These may be alternatives to reality which relate to how past events could have been different (section Thinking in Terms of Opposites in Order to Figure Out Alternatives in Everyday Life), alternative models in deductive reasoning (section Opposites and Deductive Reasoning); alternative outcomes in hypothesis testing (section Opposites and Inductive Reasoning); or alternative representations of a problem in insight problem solving (section Opposites and Insight Problem Solving). The goal of this paper is to provide evidence of the plausibility of this idea and to stimulate new directions for future research.

## Thinking in Terms of Opposites in Order to Figure Out Alternatives in Everyday Life

As psychologists have shown, the range of applications for counterfactual thinking is extensive. It supports thinking processes in various reasoning tasks (Byrne, 2016, 2017, 2018) and it is often activated when one is engaged in justifying or defending past events (Markman et al., 2008) or negative performances (Markman and Tetlock, 2000; McCrea, 2008; Tyser et al., 2012), as well as when people formulate intents or take decisions regarding future events (Markman et al., 1993; Epstude and Roese, 2008; Ferrante et al., 2013).

Although the number of counterfactual alternatives to a given event is potentially infinite, people only tend to create a limited number of alternatives. They transform exceptional events into normal events rather than vice versa (Kahneman and Tversky, 1982a), uncontrollable events become controllable (Girotto et al., 1991; Davis et al., 1995), inaction is replaced by action (Kahneman and Tversky, 1982b; Ritov and Baron, 1990), changes regarding the first events in a causal chain are prioritized over the last events (Wells et al., 1987; Segura et al., 2002) and, in contrast, the last events are changed rather than the first events in a sequence of causally independent events (Miller and Gunasegaram, 1990).

What is especially relevant to the present analysis is that no matter the content of the alternative scenario, counterfactual thinking involves imagining the opposite of what really occurred (Fillenbaum, 1974; Santamaria et al., 2005; Byrne, 2018). An example of counterfactual thinking such as “If he had caught the plane, he would have arrived on time” makes us think about both missing the plane and arriving late as opposed to catching the plane and arriving on time. “If I hadn't forgotten my umbrella, I would not be soaked to the skin now” makes us think about remembering to take the umbrella as opposed to forgetting it, and about being dry as opposed to being drenched.

Literature on the development of children has shown that by the age of 7 they are able to compare what actually occurred with alternatives to reality (e.g., German, 1999; Beck et al., 2006; Rafetseder et al., 2010) and to understand emotions based on counterfactual reasoning. That is to say that they understand how “thinking about how things could have been better” can make one feel regret, and that “thinking about how things could have been worse” can make one feel relief (see Kuczaj and Daly, 1979; Harris et al., 1996; Amsel and Smalley, 2000; Guttentag and Ferrell, 2004). Curiosity about alternative outcomes is already present in children of 4 and 5 years (e.g., Fitzgibbon et al., 2019), and can therefore be said to predate counterfactual reasoning in its strict sense. At this age, children also understand that by saying something “almost happened” (e.g., “it almost fell”) involves thinking not only about what really happened but also about the event that did *not* occur (e.g., Beck and Guthrie, 2011).

## Opposites and Deductive Reasoning

In this section, we focus on three phenomena which give an idea of the pervasiveness of opposites in deductive reasoning. Let us start with a classic example, Wason's four-card selection task (Wason, 1966, 1968). This type of task requires the ability to produce valid inferences from the information expressed in the premises. A set of four cards is placed on a table and the participants taking part in the experiment are told that each of these cards has a number printed on one side and a letter on the other side. They can, of course, only see one side of the cards, some of which are number side up and some of which are letter side up. Their task is to determine which cards need to be turned over in order to test the proposition “if there is a vowel on one side of the card then there is an even number on the other side.” In order to solve the syllogism (as required in all deductive reasoning tasks), counterexamples to the initial hypothesis need to be found. Indeed, if one single counterexample can be found, then the hypothesis has to be considered invalid, and therefore also the entire inference leading to the hypothesis is wrong (Geiger and Oberauer, 2007; Markovits et al., 2010). The first mental model that comes to mind confirms the association “vowel-even number” and people turn over the card with a vowel on it (let's call it the p-property card) and the one with an even number on it (let's call it the q-property card). However, only when the search for counterexamples fails can the initial hypothesis be confirmed. The correct procedure to follow involves testing the combination of the antecedent with the negation of the consequent (i.e., p and not-q). The frequency with which this strategy is spontaneously activated depends on the contents of the syllogism (Cheng and Holyoak, 1985; Manktelow and Over, 1991; Sperber et al., 1995; Girotto et al., 2001) and on whether the participants are given a prompt that in order to prove that a rule is true, they need to prove there is no case in which the rule is false (Augustinova, 2008). Independently of whether counterfactual thinking is spontaneously activated or prompted by a hint, in Wason's classic task we clearly see this in action since thinking “not-q” implies thinking in opposites, and “not even” numbers immediately make one think of odd numbers.

A second phenomenon in which opposites are in effect implied concerns deductive reasoning involving *scalar implicatures*. Scalar implicatures underly the interpretation of certain categorical syllogisms. Reasoners attribute an *implicit* meaning to an utterance beyond the *literal* meaning and also beyond its strict, logical meaning, based on implicit conversational pragmatic assumptions (Papafragou and Musolino, 2003; Chierchia, 2004; Papafragou and Skordos, 2016). Scalar implicatures usually involve quantifiers, but in any case, entail an interpretation along a scale of possibilities. The listener will assume that the speaker has a reason for not choosing a *stronger* term on the scale concerned. This can be clearly seen in the use of “some”—as in (1) which suggests the meaning “not all,” even though by saying “some,” logically speaking “all” cannot be excluded. Assuming that the speaker is trying to be helpful and say what he/she genuinely considers to be relevant to the conversation, the fact that he/she chooses “some” prompts the listener to think that the other person is not in a position to make an informationally stronger statement (e.g., Mary ate all of the cakes). The listener thus infers that the second statement (i.e., Mary did not eat all of the cakes) is true.

What we want to emphasize in this paper is that scalar implicatures necessarily require one to think along a scale which has opposites at each extreme. The underlying dimension in (1) goes from “all” to “none.” In the second example, the scale implied refers to the number of bears. The scalar implicature involved (“three-more than three”) is based on the contrast between “*the same* quantity” and “*a different* quantity,” which might be *more* than three or *less* than three. In this case too (assuming the speaker is trying to be helpful and say what he/she really saw and considers to be relevant), the listener infers that they saw exactly three bears and not more or less than three. The third example works in a similar way, but it plays on the contrast between “and-or,” that is, between “*both* and *only one* of the two” (Horn, 1984).

(1) a. Mary ate some of the cakes.
b. Mary did not eat all of the cakes.
(2) a. We saw three bears.
b. We did not see more than three bears.
(3) a. Elmo will buy a car or a boat.
b. Elmo will not buy both a car and a boat.

Some recent studies have focused on the role played by the cognitive structure of the scale in question in terms of pragmatic strengthening and the computation of an implicature. This idea is that the semantic structure of an adjective, for example, will systematically encourage or block certain inferences (e.g., van Tiel et al., 2016; Gotzner et al., 2018; Leffel et al., 2019). The structure of a scale is operationalized with reference to boundedness, the extremeness of the stronger pole, the nature of the weaker pole (i.e., whether it is minimum, relative, or a zero-point indicating the absence of the property), the distance between the two poles and their polarity (i.e., positive or negative). This puts constraints on the range of potential values and thereby determines the alternatives which can be used in the computation of an implicature (see also van Tiel and Schaeken, 2017).

The third phenomenon we refer to concerns reasoning in relation to logical connectives such as inclusive disjunctions (i.e., “P or Q or both”) and exclusive disjunctions (i.e., “either P or Q”). The truth table of any binary connective relating to two propositions (P, Q) holds four truth-value outcomes, in a defined order (see Table 1). The first row refers to the condition in which P is true and Q is true; the second to when P is true and Q is false; the third row to when P is false and Q is true and the fourth row to when P and Q are both false. The conditional “if P, then Q” implied in Wason's 4 card problem is only false in the second condition, that is, when P is true and Q false, which, in effect, is the condition which will solve the task.

**Table 1:** Truth table relating to propositions P and Q (for an explanation, see the main text).

First model	P	Q	Vowel	Even number
Second model	P	Not-Q	Vowel	Odd number
Third model	Not-P	Q	Consonant	Even number
Fourth model	Not-P	Not-Q	Consonant	Odd number

Many logical fallacies derive from an inappropriate interpretation of disjunctive connectives. The exclusive disjunction (“either P or Q”) is true only when the conditions described by the second and third row of the truth table hold, that is, P is true and Q is false or vice versa. Conversely, the inclusive disjunction (“P or Q or both”) is true in the first three conditions and false only in the fourth one (i.e., when P and Q are both false). In the case of a basic inference in which the major premise is an inclusive disjunction and the minor premise is affirmative (modus tollens), given the affirmation of P, reasoners tend to conclude not-Q, and given the affirmation of Q they tend to conclude not-P (e.g., Evans et al., 1993). This would be valid in the case of an exclusive disjunction, whereas we are dealing here with an inclusive disjunction. According to Robert and Brisson (2016, p. 383 ff.), and assuming the explanation of the fallacies in conditional reasoning provided by the mental model theory in terms of incomplete representations of the premises is correct (Johnson-Laird, 1983, 2010; Johnson-Laird and Khemlani, 2014), conditional fallacies all depend on the counterexample to the fallacious conclusion not being taken into account. As Table 1 demonstrates, the second, third and fourth models all imply negation, that is they refer to something which is not-P or not-Q. As stated in the introduction to this paper (section Introduction), from a cognitive point of view negation presupposes opposition. In the examples in Table 1, not-even means odd, not-vowel means a consonant. However, not all domains are conceptualized as being mutually exclusive. The binary or graded structure of a dimension (e.g., Kennedy and McNally, 2005) substantially influences the amount of shift which spontaneously comes to mind. The semantic meaning of the sentence *John is not handsome* merely entails that John is something less than handsome, that is, he might be attractive, average looking or even ugly, depending also on other contextual and pragmatic factors (e.g., Paradis, 2008). Despite the fact that this is a matter of modulation, there is in any case a presupposed reference to opposition.

## Opposites and Inductive Reasoning

Hypothesis testing epitomizes the process of inductive reasoning (Oaksford and Chater, 1994; Vartanian et al., 2003). It underlies not only scientific reasoning (Mahoney and DeMonbreun, 1977; Langley et al., 1987; Klahr and Dunbar, 1988), but also many classes of human judgments, including social inferences regarding individual or group behaviors (e.g., Nisbett and Ross, 1980; DiDonato et al., 2011). The process of hypothesis testing involves forming hypotheses, and then gathering evidence in order to test and revise these hypotheses (e.g., Klayman and Ha, 1987; Heit, 2000; Evans, 2007).

Wason's rule discovery task (Wason, 1960) represents the standard paradigm employed to study hypothesis testing behavior. In this task, the participants are asked to discover the arithmetic rule devised by the experimenter that applies to a series of three number sequences. They are given an initial sequence (2-4-6) that fits in with the rule and are invited to construct their own series of three number sequences in order to test any hypotheses they formulate about the rule. The experimenter provides feedback and once the participants feel confident that they have found the rule, they announce their finding. Generally, the process goes on until the participants come up with the right rule or it is stopped after a fixed amount of time. This task requires the reasoners to create not only series of numbers that confirm their idea regarding the rule, but also series of numbers which disconfirm the rule (Rossi et al., 2001; Evans, 2007). Most people do not proceed in this way, and this task illustrates how people generally tend to follow a confirmation bias in hypothesis testing tasks. In fact, only around 20% of participants find the correct rule at the first attempt (Wason, 1960; Farris and Revlin, 1989b). Analyses of the reasoning process used by those participants who find the correct rule at the first attempt revealed that they applied a counterfactual strategy both when generating hypotheses and when testing them (Klayman and Ha, 1987; Farris and Revlin, 1989a,b; Oaksford and Chater, 1994; Gale and Ball, 2012). Counterfactual hypotheses were formed by varying one thing at a time; for instance, if the initial hypothesis was “any series of three even numbers in increasing order”, participants created a counterfactual hypothesis such as “any series of three odd numbers in increasing order” or “any series of three even numbers in decreasing order” or “any series of three numbers which are the same” (Tschirgi, 1980). The examples not only make it clear that each of the hypotheses transforms and tests the effect of making one transformation at a time, but also show that the transformations are based on opposites (i.e., even is transformed into odd, increasing is transformed into decreasing and different numbers are transformed into the same numbers).

There has been a great deal of debate, in some way inspired by Popper (2005) falsification model, on the exclusive need to adopt disconfirmatory strategies in inductive inference tasks (e.g., Farris and Revlin, 1989a,b, 1991; Gorman, 1991) and various attempts have been made to train participants to use disconfirmatory strategies on the 2-4-6 rule discovery task or in other similar tasks. One of these is the ‘Eleusis' card game (first invented by Robert Abbott in1956). The problem is essentially very similar to Wason's triples task. The game involves one player (the dealer) dealing a row of cards in sequence and this person then chooses a secret rule to determine which card or cards can be played subsequently. The other players take turns to try and guess the rule for the sequence by placing one or more cards on the table. The dealer says whether these cards fit in with the rule or not. Gorman et al. (1984) trained participants to apply either a confirmatory strategy (“test your guesses by concentrating on playing cards that will be correct”) or a disconfirmatory strategy (“test your guesses by deliberately playing cards that you think will be wrong”), or a mixed strategy (“first, concentrate on getting right answers until you have a guess; then test your guess by deliberately playing cards you are sure will be wrong”). Similar strategies were tested by Tweney et al. (1980), but using the 2-4-6 problem. Gorman et al. (1984) found that groups using a disconfirmatory strategy found the correct rule 72% of the time, those applying a combined strategy were correct 50% of the time and those using a confirmatory strategy were only right in 25% of cases. Tweney et al. (1980) found that a disconfirmatory strategy was easily induced, but it did not lead to greater efficiency and neither did a mixed strategy. A comparison of the two studies suggests that a critical element is whether participants are given feedback on whether their guesses are consistent with the rule or not. Indeed, in Gorman and Gorman's study Gorman and Gorman's 1984, the participants were allowed to make as many guesses as they liked within a half hour time limit and playing a maximum of 60 cards. They were requested to write down their guesses, the number of the card and the time at which they made the guess, but received no feedback until the end of the experiment.

Improvements in performance were generally found when the participants were prompted to work on the discovery of two interrelated rules (this condition is known as dual goal instruction). Success rates at the first attempt typically rise to over 60% in the dual goal instruction condition, when complementary rules are considered (e.g., Tweney et al., 1980; Tukey, 1986; Gorman et al., 1987; Wharton et al., 1993). Why does this type of manipulation make a difference? Gale and Ball (2006, 2009) hypothesized and subsequently verified that a critical element is whether the two rules stimulate participants to think in terms of contrast classes, that is, in term of relevant oppositional contrasts. As confirmed in a further study on finding rules for triple sequences (Gale and Ball, 2012), the facilitatory effect of the dual goal instruction was greater when the participants were given the 6-4-2 triple sequence as an exemplar of the second rule (with a success rate of around 75%), than when the exemplar was 9-8-1 (with only a 36% success rate) or the 4-4-4 sequence (with a 20% success rate). The 6-4-2 triple makes the relevant contrast immediately evident; the 9-8-1 triple contrasts in more than one dimension and thus only to some extent shifts the participants' attention toward the critical characteristics while the 4-4-4 triple suggests a contrast class (same vs. different numbers) which is not useful in any way.

The idea of a “contrast class” refers here to a psychological rather than logical concept (Oaksford, 2002, p. 140). It does not merely identify the logical complement set. In Oaksford's example, the sentence “Person X is not drinking coffee”, entails a logical complement set which includes all beverages except coffee (i.e., whisky, cola, or sparkling water etc.), but the immediate hypothesis that comes to people's minds is that the person must be drinking some other hot beverage such as tea. Thus, we see that the identification of a contrasting set is driven by cognitive “relevance” (Sperber and Wilson, 1986/1995) which means that reasoners focus on one or more relevant contrasts out of a range of alternatives. These in turn might depend on various aspects such as perceptual relevance, semantic factors, and also contextual aspects (e.g., situational, socio-linguistic, stylistic, or prosodic factors). These influence what the reasoner may decide constitutes a contrast. The identification of a contrast tends to be more straightforward if there is clearly a binary opposite, for example, even-odd, ascending-descending, or vowel-consonant (for a discussion of the concept of relevance in the identification of contrast sets, see Tenbrink and Freksa, 2009, but also Paradis et al., 2009 for an analysis of the more general issue of opposites and canonicity). We return to this point in the final discussion.

In line with the main claim of this paper, we suggest that an explanation for why stimulating reasoners to think in opposites by means of facilitatory hints involving contrast classes seems to be more effective than prompting them to apply disconfirmatory strategies (e.g., Tweney et al., 1980) might depend on the fact that this strategy does not ask people to focus on testing hypotheses they expect to be wrong. In contrast, it allows them to make a positive search using opposites to identify potential falsifiers of the rule. This is not very different to Gale and Ball's suggestion (Gale and Ball's 2012, p. 416–417) which is, however, based on Oaksford and Chater's (1994) iterative counterfactual model.

## Opposites and Insight Problem Solving

Another domain in which thinking in terms of opposites is crucial is problem solving. The process of problem solving starts off from an initial state, such as a given situation or problem statement; the solver works toward the goal state (i.e., the solved problem) passing through various intermediate states along the way. *Insight* problems cannot be solved by means of the mere application of predefined rules and they are typically characterized by a moment when the solution arrives suddenly and unexpectedly (often known as the “Aha!” moment) after an impasse, usually as part of a stadial process (Ohlsson, 1992; Öllinger et al., 2014; Fedor et al., 2015). It is crucial that before this revelatory moment there has been some form of representational change which has allowed the person to overcome a blockage deriving from a former (unproductive) representation of the problem (Knoblich et al., 1999; Öllinger et al., 2008; Ohlsson, 2011) and various studies have focused on the mechanisms underlying this change and on how to facilitate it (see Gilhooly et al., 2015).

The relevance of opposites in this representational change was first theorized by the Gestalt psychologists Duncker (1945) and Wertheimer (1945/1959). According to Wertheimer, solving a problem means creating new groupings within the overall structure of the problem, by unifying the elements of the problem that were initially separated and dividing elements that were initially unified thereby creating a new mental organization of the problem (Wertheimer, 1945/1959). Duncker (1945) pointed out that what is needed in this restructuring is a shift of function in the elements within a system, and he explicitly defined this shift in terms of opposites. In the last 10 years, a number of studies have been conducted that offer direct or indirect evidence that thinking in opposites supports a relaxation of the constraints that prevent the reasoners from seeing the solution. For example, in the ping-pong ball problem (Ansburg and Dominowski, 2000), reasoners are asked to work out how to throw a ping-pong ball (without bouncing it off any surface or tying it to anything) so that it will travel a short distance, come to a dead stop and then roll back on its tracks. In order to reach the solution, it is necessary to imagine the ball following a vertical trajectory, but this contrasts with the mental model which initially comes to peoples' minds which contains the implicit assumption of a horizontal trajectory (Murray and Byrne, 2013). This bias toward a horizontal trajectory is the cause of the impasse. The problem solver needs to stop focusing on a horizontal trajectory and start thinking of a vertical trajectory in order to resolve the impasse.

**Figure 3:**
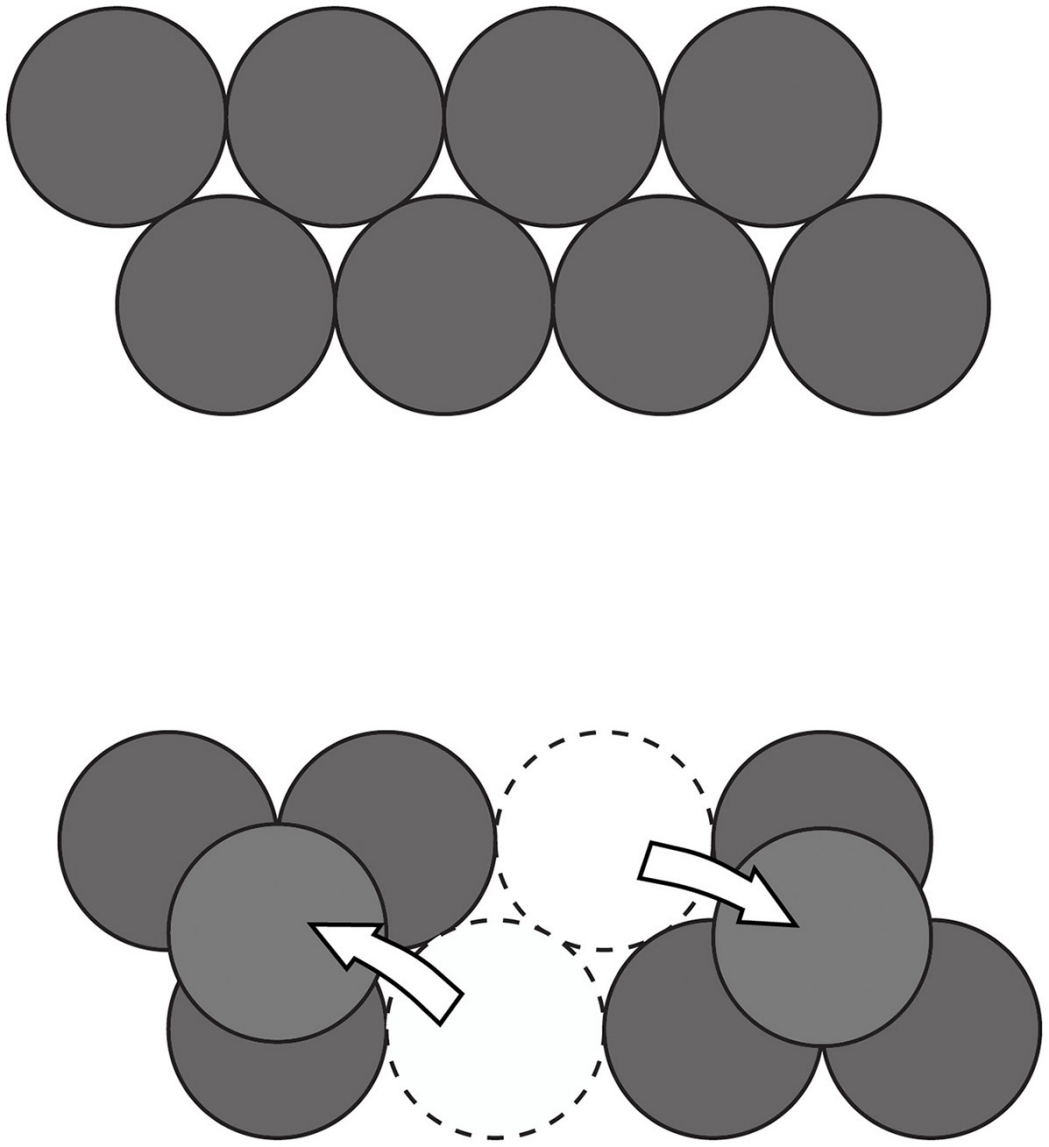
Initial configuration (**Top** image) and final configuration (**Bottom** image) in the eight coin problem (Ormerod et al., 2002). The arrow represents the moves which need to be made.

Explicitly stimulating participants (either by means of training or using hints) to explore the initial structure of a problem in terms of its salient spatial features and to systematically transform them into their opposites has proved to be effective in a series of studies (e.g., Branchini et al., 2015a,b, 2016; Bianchi et al., 2020). To exemplify the point, let's consider the eight coin problem in which the participants are asked to start from a given configuration of eight disks (see the top image in Figure 3), and to move only two coins in such a way that the new arrangement will respect the condition that each coin only touches three other coins. The moves which need to be made and the final solution are represented in the bottom image in Figure 3. If one looks at the initial configuration, one notices various aspects: that the configuration is oriented *horizontally*; that there is the *same* number of coins in each of the two rows; that they are *misaligned*; that they are *united* to form a single group of coins and that they lie on the same plane (i.e., it is a *bidimensional* configuration). Encouraging the participants to focus on the properties they identify while exploring the structure of the problem and then to think of these in terms of their opposites (i.e., *horizontal-vertical, equal-different number, aligned-misaligned, united-separated* and *bidimensional-three dimensional*) helped the participants to ascertain which aspect they needed to transform, increased the number of attempts made and led to a better performance. In the eight coin problem, the two pairs of opposites which are functional are *united-separated* (since it is necessary to split the group of coins into two separate subgroups) and *bidimensional-three dimensional* (since one needs to position two of the coins *over* the other three so that they are then superimposed and no longer coplanar).

## Discussion

In this paper, we have reviewed studies carried out in the last 20 years to explore the idea that opposition is a general organizing principle for the human mind. In addition to the research revised in the introduction (section Introduction) which emphasized the importance of opposites as a basic structure for human perception, human language, and relational reasoning, in this paper we put forward the idea that they are also a pervasive structure which is implied in various reasoning tasks. We have shown that “thinking in opposites” is fundamental to how people naturally think when they imagine alternatives to reality in everyday counterfactual reasoning (see section Thinking in Terms of Opposites in Order to Figure Out Alternatives in Everyday Life), that it is presupposed in various deductive reasoning phenomena, from understanding scalar implicatures to counterfactual reasoning in basic conditional reasoning, as well as in reasoning about disjunctive connectives (see section Opposites and Deductive Reasoning), that it supports inductive thinking in that it is involved in the identification of contrast class sets which are crucial to hypothesis testing (see section Opposites and Inductive Reasoning) and that it upholds representational change in insight problem solving thereby paving the way to the resolution of the problem (see section Opposites and Insight Problem Solving). In this sense this overview might lead to the impression that we do, in fact, use opposites more than we think.

On the other hand, as part of the picture which emerged from the review of the literature discussed in this paper, there is the consideration that counterfactual thinking is spontaneously used in some circumstances in everyday life, but is not so often spontaneously activated in inductive and deductive reasoning, and neither is it used as a purposeful strategy in problem solving. However, various studies investigating possible ways to stimulate reasoners to overcome typical reasoning biases in all of these domains suggest that giving them a hint to “think in opposites” points them in the right direction and improves their performance. In this sense opposites are used less than they could be. The facilitatory effect of using opposites as a strategy has been found in some studies on people's performance in classic deductive tasks (e.g., Augustinova, 2008), in Wason's triple inductive task (e.g., Gale and Ball, 2009, 2012), and in visuo-spatial insight problem solving (e.g., Murray and Byrne, 2013; Branchini et al., 2015a,b, 2016; Bianchi et al., 2020).

The tasks and prompts used in the abovementioned studies are very different from each other and are also very specific. Further experimentation is therefore needed before any conclusions suggesting that encouraging people to think in terms of opposites kick starts an intuitive strategy that enhances their performance can be generalized beyond the conditions of validity tested thus far. For instance, Branchini et al. (2015a, 2016) tested the effects of implicit hints and explicit training programs based on thinking in opposites in classic visuo-spatial insight problems. They found that this was effective both in terms of modifying the contents of the attempts made (i.e., the choice of properties to focus on), and in terms of the success rate. This has, however, not been tested with verbal insight problems (such as those studied, for instance, by Dow and Mayer, 2004; Macchi and Bagassi, 2015; Patrick et al., 2015), and neither has an adaptation of the training been tested in a hypothesis testing condition, such as for example Wason's triplets condition. If the seed triple 2-4-6 in Wason's problem leads to the hypothesis that the rule involves an “ascending series of even numbers, regularly increasing by two,” an explicit prompt to think in opposites would immediately suggest the precise direction in which to search, that is, testing whether the series is descending rather than ascending, whether it is made up of odd rather than even numbers, or whether it has irregular rather than regular intervals between the numbers, and so on.

Another aspect that future research might help to clarify concerns the relation between thinking in opposites and Type 1 and Type 2 processes as defined by Evans and Stanovich (2013a,b). Type 1 processes refer to fast, unconscious, automatic processing, Type 2 to slow, conscious, controlled processing (see also Sloman, 1996; Kahneman, 2011; Stanovich, 2011). The various different types of prompts used in the studies revised in this paper involved, in fact, both implicit and explicit suggestions. In the studies carried out by Augustinova (2008); Gale and Ball (2012); Murray and Byrne (2013) and Branchini et al. (2015a), implicit hints were enough to improve the participants' performance. These consisted of, respectively, a falsification cue, a contrast class cue, a counterexample, and an invitation to list the features of the problem and their opposites. There was no explanation as to why this would help. In these studies, the facilitating factors are implicit processes (which hinge on Type 1 processes) since the participants were exposed to the hints without any awareness of how they could be useful. In other studies from which a facilitatory effect emerged (see the studies done by Branchini et al., 2016; Bianchi et al., 2020), the participants were explicitly trained to use thinking in opposites as a strategy. This represents an analytically conscious suggestion which hinges on Type 2 processes. Based on these results, a provisional hypothesis that a prompt might be effective on both levels, within specific limits, seems to emerge, but further studies are of course needed to consolidate or disprove this.

Another aspect that deserves further investigation concerns whether this strategy is effective both when individuals are working alone or in groups. Content related hints to use opposites turned out to be effective in individual settings in the studies done by Gale and Ball (2012) and Murray and Byrne (2013), whereas positive effects resulting from general training to think in opposites were found in small group settings in the studies carried out by Bianchi et al. (2020) and Branchini et al. (2015a, 2016). Augustinova also worked with groups (Augustinova et al., 2005; Augustinova, 2008). Does this mean that more generic prompts suffice in group contexts whereas a content specific prompt is needed in individual contexts? Whether this relates to the processing dynamics which are natural in groups but are not present in the case of individual reasoners (Tindale and Kameda, 2000; Tindale et al., 2001) is an aspect which is worth investigating further, taking into account, however, the fact that it is always easier to contradict another person's best guess than it is to question one's own best guess (Poletiek, 1996).

Notably, as mentioned in the introduction to this paper, the strength of a prompt to generically “think in opposites” is based on the intuitive nature of opposition that it presupposes, as compared to more complex technical or logical definitions of opposition (i.e., counterfactuality, antynomy, and logical opposition). This means that it can *easily become* a deliberate strategy to produce systematic manipulations of an initial problem in order to resolve it. Moreover, since opposites, by definition, consist of pairs of properties, they offer a method of opening up the space within which a search is carried out, while at the same time giving precise directions. This combination of openness and boundary shifting fits in with the requisites of an effective cognitive heuristic according to, for instance, Öllinger et al. (2013). Therefore, opposites allow one to think not only in terms of not-x, but also in terms of alternatives which are clearly identifiable since they lie along well-defined dimensions. Contrasts are not set in stone. What makes a contrast relevant depends on contextual aspects, the mental framework, and various linguistic and pragmatic conditions (e.g., Paradis et al., 2009; Tenbrink and Freksa, 2009). This strategy, therefore, is one which is adaptive and flexible, and the variability of alternatives that come up is extremely wide, if not infinite.

The dual nature of opposition also conforms well with the duality that is inherent to the type of thinking implied in scientific discoveries, as discussed by Platt (1964) in relation to paramount discoveries in Molecular Biology and Physics in a short but extremely rich article published in the *Science* journal. He bases his claim on a variety of remarkable examples which demonstrate that the most efficient way for humans to use their minds when solving scientific questions consists of, at each step, explicitly setting down the question and the alternative hypotheses before conducting crucial experiments in order to exclude some alternatives and then adopt what remains. This procedure is known as strong inference and it is clearly modeled in terms of a decision tree (a conditional inductive trees or a logical tree) in which every time the branches fork, we can choose to go right or left. Thus, we can reasonably say that opposites, as conceptualized in this paper, seem to be the cognitive mechanism underlying the identification of these forks in the branches. “Thinking in opposites” is a way of thinking which is within everyone's reach and can easily become a deliberate thinking strategy. Due to the fact that the identification of opposites is sensitive to situations, this strategy can potentially lead to the creation of an extremely complex, rich set of alternatives for reasoners to test systematically.

## Author Contributions

EB, EC, RB, US, and IB contributed to conception of the review and wrote sections of the manuscript. EB and IB wrote the first draft of the manuscript. All authors contributed to manuscript revision, read, and approved the submitted version.

## Conflict of Interest

The authors declare that the research was conducted in the absence of any commercial or financial relationships that could be construed as a potential conflict of interest.

## Publisher's Note

All claims expressed in this article are solely those of the authors and do not necessarily represent those of their affiliated organizations, or those of the publisher, the editors and the reviewers. Any product that may be evaluated in this article, or claim that may be made by its manufacturer, is not guaranteed or endorsed by the publisher.
